# Prediction of Cervical Lymph Node Metastasis in Clinically Node-Negative T1 and T2 Papillary Thyroid Carcinoma Using Supervised Machine Learning Approach

**DOI:** 10.3390/jcm12113641

**Published:** 2023-05-24

**Authors:** Marina Popović Krneta, Dragana Šobić Šaranović, Ljiljana Mijatović Teodorović, Nemanja Krajčinović, Nataša Avramović, Živko Bojović, Zoran Bukumirić, Ivan Marković, Saša Rajšić, Biljana Bazić Djorović, Vera Artiko, Mihajlo Karličić, Miljana Tanić

**Affiliations:** 1 Department of Nuclear Medicine, Institute for Oncology and Radiology of Serbia, 11 000 Belgrade, Serbia; 2 Faculty of Medicine, University of Belgrade, 11 000 Belgrade, Serbia; 3 Center for Nuclear Medicine with PET, University Clinical Center of Serbia, 11 000 Belgrade, Serbia; 4 Faculty of Medical Sciences, University of Kragujevac, 34 000 Kragujevac, Serbia; 5 Department of Power, Electronics and Telecommunications, Faculty of Technical Sciences, University of Novi Sad, 21 000 Novi Sad, Serbia; 6 Institute of Medical Statistics and Informatics, Faculty of Medicine, University of Belgrade, 11 000 Belgrade, Serbia; 7 Surgical Oncology Clinic, Institute for Oncology and Radiology of Serbia, 11 000 Belgrade, Serbia; 8 Department of Anesthesiology and Intensive Care Medicine, Medical University Innsbruck, 6020 Innsbruck, Austria; 9 School of Electrical Engineering, University of Belgrade, 11 000 Belgrade, Serbia; 10 Department of Experimental Oncology, Institute for Oncology and Radiology of Serbia, 11 000 Belgrade, Serbia; 11 UCL Cancer Institute, London WC1E 6DD, UK

**Keywords:** papillary thyroid carcinoma, lymph node metastasis, machine learning

## Abstract

Papillary thyroid carcinoma (PTC) is generally considered an indolent cancer. However, patients with cervical lymph node metastasis (LNM) have a higher risk of local recurrence. This study evaluated and compared four machine learning (ML)-based classifiers to predict the presence of cervical LNM in clinically node-negative (cN0) T1 and T2 PTC patients. The algorithm was developed using clinicopathological data from 288 patients who underwent total thyroidectomy and prophylactic central neck dissection, with sentinel lymph node biopsy performed to identify lateral LNM. The final ML classifier was selected based on the highest specificity and the lowest degree of overfitting while maintaining a sensitivity of 95%. Among the models evaluated, the k-Nearest Neighbor (k-NN) classifier was found to be the best fit, with an area under the receiver operating characteristic curve of 0.72, and sensitivity, specificity, positive and negative predictive values, F1 and F2 scores of 98%, 27%, 56%, 93%, 72%, and 85%, respectively. A web application based on a sensitivity-optimized kNN classifier was also created to predict the potential of cervical LNM, allowing users to explore and potentially build upon the model. These findings suggest that ML can improve the prediction of LNM in cN0 T1 and T2 PTC patients, thereby aiding in individual treatment planning.

## 1. Introduction

Papillary thyroid carcinoma (PTC) has emerged as the most common thyroid malignancy over the last thirty years [1,2]. Whereas the majority of patients with PTCs generally have an excellent prognosis, those with the cervical lymph node metastasis (LNM) may have an elevated rate of local recurrence [3,4]. Therefore, it is crucial to establish the cervical lymph nodes’ metastatic status in order to adequately stage the disease and plan appropriate treatment management.

In conjunction with a total thyroidectomy, therapeutic cervical lymph node dissection (LND) is indicated when a central or lateral nodal disease is clinically evident based on pretreatment physical examination and radiological workup [5]. Nonetheless, managing cervical LNM in patients with small PTC (T1 or T2) who do not have clinically evident nodal metastases (cN0) has been the subject of controversy [3,6,7,8]. For such PTCs, different approaches are currently proposed across guidelines and scientific reports such as clinical follow-up involving therapeutic neck dissection reserved in the subsequent development of LNM, prophylactic central LND, and sentinel lymph node biopsy (SLNB) [2,9,10,11,12]. However, all these strategies have certain advantages and disadvantages, being the subject of discussion. For instance, while offering the potential advantage of decreasing disease recurrence, prophylactic central LND is accompanied by potential morbidity and may worsen overall quality of life [13,14]. Conversely, although SLNB is less invasive as it removes the need for unnecessary prophylactic neck dissection, it has not been widely adopted due to technical issues [15,16].

Although preoperative ultrasound (US) and computed tomography (CT) are valuable imaging techniques in diagnosing nodal metastasis, roughly 40% of cN0 patients may still have undetected LNM [15]. Therefore, establishing PTC risk factors is beneficial to identify those patients carrying a higher risk of nodal disease and therefore require prophylactic LND and possible postoperative radioactive iodine (RAI) treatment [17]. At the same time, care must be taken to avoid the morbidity of neck dissection in patients without nodal metastasis. As opposed to the “one size fits all” approach recommended by the guidelines, improved methods are necessary in order to choose optimal treatment based on individual patient characteristics [2,6,9].

Several predictive models have been used to estimate the presence of LNM in patients with PTC, including the recently introduced machine learning (ML) approach, which has the potential to significantly advance this field [6,18]. Machine Learning (ML) is a branch of artificial intelligence (AI) focusing on applying algorithms able to identify patterns in historical data. These are further used to make predictions on new unseen data [19]. Since ML is both able to recognize nonlinear relationships in the data and complex interactions among multiple predictors, it may potentially outperform conventional statistical methods in LNM prediction [20]. A recent study conducted by Zhu et al. (2021) demonstrated that ML classifiers are capable of predicting lymph node metastasis in cN0 papillary thyroid carcinoma patients based on clinicopathological parameters [6]. However, their work was focused only on detecting central lymph node metastasis, although a significant number of lateral LNMs may remain after surgery and present as recurrence [4]. Therefore, this study aimed to develop, compare, and validate four ML classifiers for the prediction of both central and lateral LNM by using simple clinical and histopathological data. We hypothesized that the supervised ML classifier may enable more precise patient follow-up, leading to the earlier detection of LNM and adequate postsurgical treatment. 

## 2. Materials and Methods

### 2.1. Patient Selection and Data Collection

**Figure 1:**
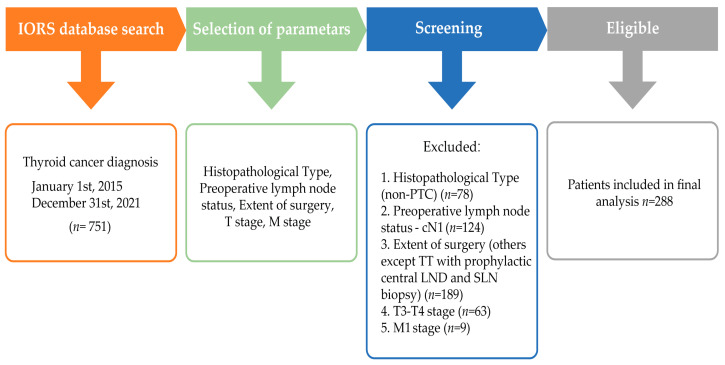
Consort diagram of the study. Abbreviations: IORS—Institute for Oncology and Radiology of Serbia; T stage—the size of the tumor and any spread of cancer into nearby tissue; M stage—presence or absence of distant metastases; PTC—papillary thyroid carcinoma; TT—total thyroidectomy; LND—lymph node dissection; SLN—sentinel lymph node.

We retrospectively reviewed the medical records of thyroid cancer patients surgically treated between January 2015 and December 2021 at the Institute for Oncology and Radiology of Serbia (IORS). Patients were included if they had histologically confirmed PTC no larger than 40 mm, staged as T1 or T2. All the eligible patients showed no evidence of clinically palpable and radiologically confirmed lymph node metastases (cN0). We excluded patients who had: (1) non-PTC histology, (2) clinical evidence of LNMs (cN1), (3) distant metastasis, and (4) patients who did not undergo total thyroidectomy with prophylactic CLND and SLNB of the lateral neck compartment. The patient selection process is shown in Figure 1. The study was approved by the Medical Ethics Committee of the School of Medicine, University of Belgrade (#61206-1165/2-22).

The demographic and clinical characteristics of interest were: age, sex, thyroid stimulating hormone (TSH) value, tumor size, multifocality (number of tumor foci), bilaterality, microscopic extrathyroidal extension (micro ETE), as well as thyroid capsular invasion (CI) and lymphovascular invasion (LVI). The initial staging of the tumor was reevaluated according to the 8th edition of the American Joint Committee on Cancer (AJCC) Staging System [21]. The endpoint for this study was the presence or absence of LNM based on a histopathologic evaluation of the cervical lymph nodes.

### 2.2. Surgical Procedure

All included patients have been treated with a total thyroidectomy and prophylactic central LND. In order to identify any occult lateral LNM and determine the need for a selective lateral LND, a sentinel lymph node biopsy of the lateral neck compartments was conducted.

For the surgical procedure, 0.2–0.5 mL 1% methylene blue dye was injected peritumorally just below the thyroid capsule. The capsule was then coagulated to prevent dye leakage. If there was a bilateral tumor, the procedure was also performed on the other lobe. Further, the lobe containing the nodule suspicious of thyroid cancer was removed and sent to a frozen section study (FSS) to confirm PTC. After histopathologic verification, subsequent completion of thyroidectomy was performed along with prophylactic central LND (level VI). The lateral neck compartments were investigated for blue-stained lymph nodes which were defined as the sentinel lymph nodes (SLNs). If no colored lymph nodes were located, the lymph node of the colored afferent lymphatic was considered to be the SLN. Sentinel lymph nodes were then removed and subjected to the FSS. For metastatic SLNs, a concurrent LND was immediately performed [22].

### 2.3. Development of Machine Learning Classifiers

A total of eight variables containing both PTC patients’ demographic and clinicopathological characteristics (Table 1) were used to develop ML-based classifiers to predict LNM. The following four ML classifiers were applied in this study: k-Nearest Neighbor (k-NN), Support Vector Machines (SVM), Decision Tree (DT), and Logistic Regression (LR).

Due to the data being collected retrospectively, any missing data for continuous variables were processed under median imputation, keeping only the variables with less than 10% of missing values. The patients were randomly divided into two sets under a 70:30 distribution where the ML algorithm was trained using 70% of patients (*n* = 201) and tested through the application of the remaining 30% of patients (*n* = 87). To avoid possible model bias, a stratification was performed to ensure that lymph node metastases cases were equal in frequency to those without LNM in both sets (Table S1). 

The training set was used for model comparison and development. A 10-fold cross-validation (CV) was applied in the training group in order to assess the true prediction error and degree of overfitting. All ML classifiers were compared under the broad spectrum of their individual corresponding configurations (Figures S1 and S2). For the kNN classifier, we compared 15 distance measures including Euclidean, Manhattan, Chebyshev, Minkowski, Hamming, Canberra, Bray–Curtis, Jaccard, Matching coefficient, Dice, CityBlock, Rogers–Tanimoto, Russell–Rao, Sokal–Michener, and Sokal–Sneath. For the SVM classifiers, we tested polynomial, radial basis function (RBF), and sigmoid kernel types. The solvers were tested for the LR model—including the liblinear solver, the Limited-memory Broyden–Fletcher–Goldfarb–Shanno (L-BFGS) algorithm, and the Newton-conjugate gradient method (Newton-CG)—whose performances were then compared. Finally, we trained a single decision tree model and increased its depth until overfitting occurred, to determine the optimal depth for the data. 

**Figure 2:**
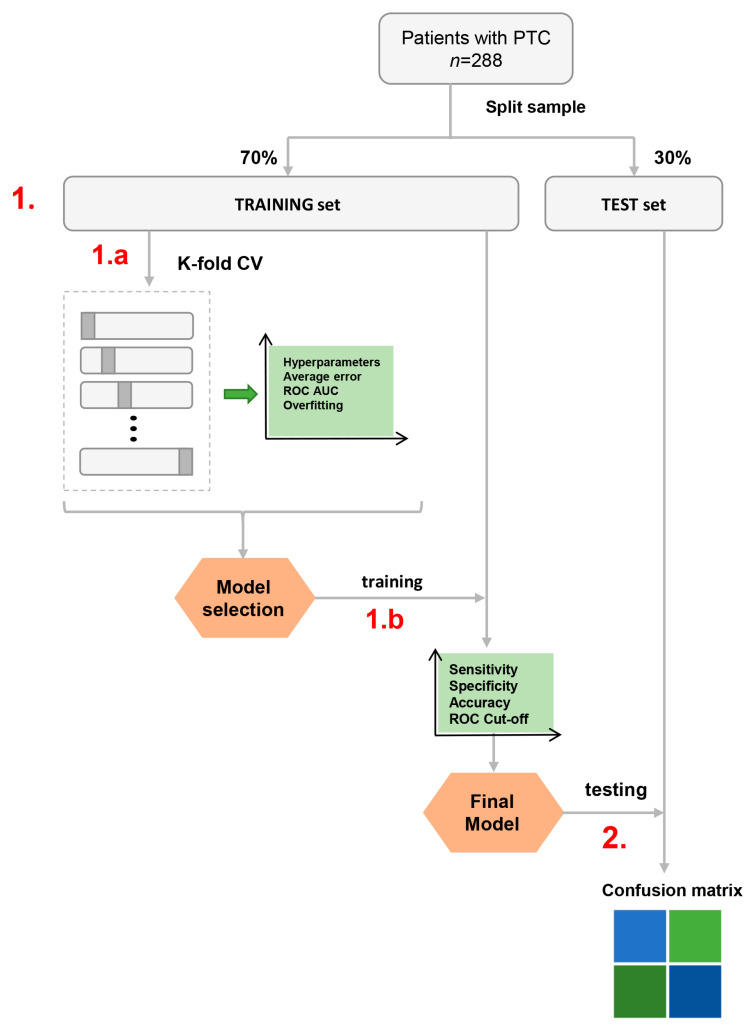
The classifier building outline. 1: Classifier-training set; 1a: k-fold cross-validation (CV)- model hyperparameter selection based on average error rate, average AUC (area under the receiver operating characteristic curve (ROC)), overfitting plots; 1b: model training (on the whole training set) and cutoff selection based on sensitivity, specificity and, ROC. 2: Classifier evaluation on the test cohort.

The area under the receiver operating characteristic curve (AUC), prediction error, sensitivity, and specificity plots were assessed to select the model with the highest predictive performance AUC, minimal error, and overfitting. The selected ML classifier with optimal settings was then fine-tuned and applied to the entire training data set. Thereafter, the final model’s cutoff was selected prioritizing the highest sensitivity by which patients with LNM may be discriminated from dose without lymph node metastases. This level of cutoff was intended to identify individuals with a high probability of LNM indicating the need for additional treatment or more tailored, patient-orientated follow-up. The test set was then used to assess the performance of the final ML classifier. The classifier building outline is shown in Figure 2.

### 2.4. Statistical Analysis and Software 

Descriptive methods (frequencies, percent, mean, standard deviation (SD), median and range) were used to summarize the data. For normal distribution data testing, the Shapiro–Wilk test was used. For comparison of demographic and clinicopathological characteristics among different patient subgroups, the Wilcoxon rank-sum, Pearson chi-square, Student’s *t*-test, and Fisher exact tests were used. The two-tailed statistical significance level was set at *p* < 0.05. All statistical analyses were performed with the IBM SPSS Statistics 22 software (SPSS Inc., Chicago, IL, USA).

The ML algorithms were implemented using Python (version 3.9.6) and various libraries, including numpy (version 1.21.2), pandas (version 1.3.2), seaborn (version 0.11.2), matplotlib (version 3.4.3), and scikit-learn (version 0.24.2). A web application was developed using the Python web framework Flask, and the final ML model was deployed using the joblib module. The performances based on the confusion matrix were used to compare the different models on the test set. True positive (TP), true negative (TN), false positive (FP), and false negative (FN) rates were assessed. These parameters were further used to obtain sensitivity, specificity, negative predictive value (NPV), positive predictive value (PPV), accuracy, and F1 and F2 scores under the Bayes theorem based on LNM prevalence in our cohort (*p* = 49%). Shapley values, which represent a feature’s contribution to the model for a specific patient, were computed using the Python SHAP module (version 0.41.0) to explain the final model’s variable weights. Shapley values, when linear, are equal to the weight of the feature in the model multiplied by the feature’s value. When non-linear, Shapley values represent a feature’s contribution in relation to all other features of the model [23].

## 3. Results

### 3.1. Descriptive Statistics

Demographic and clinicopathological characteristics of patients are provided in Table 1. We included 288 PTC patients, of whom 72 were males (25%) and 216 females (75%) with an average age of 47.0 ± 13.5 years. The median tumor diameter was 10 mm, ranging from 1 mm to 40 mm. More than 50% of patients (54.2%) presented with papillary micro-carcinomas. Cervical LNM was confirmed histopathologically in 141 cases (49%). More specifically, 69 (24%) out of all patients had isolated central lymph node metastases. Lateral and central LNM were simultaneously presented in 50 (17.4%) patients while 22 (7.6%) patients were observed to have skip metastasis (presence of lateral LNM without central lymph node involvement).

**Table 1:** Summary description of the original dataset.

Charasteristics	Total (*n =* 288)
Age	47.0 ± 13.5
Sex	
Male	72 (25.0)
Female	216 (75.0)
TSH value (µIU/mL)	1.70 (0.01–11.9)
Tumor size (mm)	10 (1–40)
Tumor size (categories, mm)	
≤5	57 (19.8)
6–10	99 (34.4)
11–20	86 (29.9)
21–40	46 (16.0)
Multifocality	
No	184 (63.9)
Yes	104 (36.1)
Number of tumor foci	
1	184 (63.9)
2	62 (21.5)
≥3	42 (14.6)
Bilateral	
No	212 (73.6)
Yes	76 (26.4)
Thyroid capsular invasion or micro ETE	
No thyroid capsular invasion or micro ETE	181 (62.8)
Thyroid capsular invasion	58 (20.1)
Micro ETE	49 (17.1)
Lymphovascular invasion	
No	273 (94.8)
Yes	15 (5.2)
LNM	
No LNM	147 (51.0)
Central LNM only	69 (24.0)
Lateral LNM only	22 (7.6)
Central and lateral LNM	50 (17.4)

Values are presented as median (minimum–maximum), mean (standard deviation), or number (%) of patients. Abbreviations: TSH—thyroid stimulating hormone; micro ETE—microscopic extrathyroidal extension; LNM—lymph node metastasis.

### 3.2. Univariate Analyses 

Univariate analyses identified LNM risk factors in patients with PTC. A comparison of the characteristics between LNM and non-LNM patients is given in Table 2. Younger patients were at an increased risk for lymph node metastases (*p* < 0.001). Patients with LNM presented tumors larger in size compared to non-LNM patients (*p* < 0.001). Patients who had no metastases most commonly presented as micro-carcinoma (73.5%). The occurrence of LNM was also associated with multifocality and bilaterality (*p* < 0.05). Upon investigating the number of tumor foci, it showed that LNM increased according to the number of tumor foci (*p* = 0.004). Tumor capsular invasion and microscopic extrathyroidal extension were found to be associated with an increased risk of LNM (*p* < 0.001). Lymphovascular invasion was also correlated to LNM (*p* < 0.05). There was no significant difference between LNM-positive and LNM-negative patients in terms of patient sex and preoperative TSH value.

### 3.3. Performance Metrics for the ML Classifiers

To select the optimal ML classifier for predicting patient outcomes, we compared the performances of four ML models (k-NN, SVM, LR, and DT) using a range of their individual configurations. 

**Figure 3:**
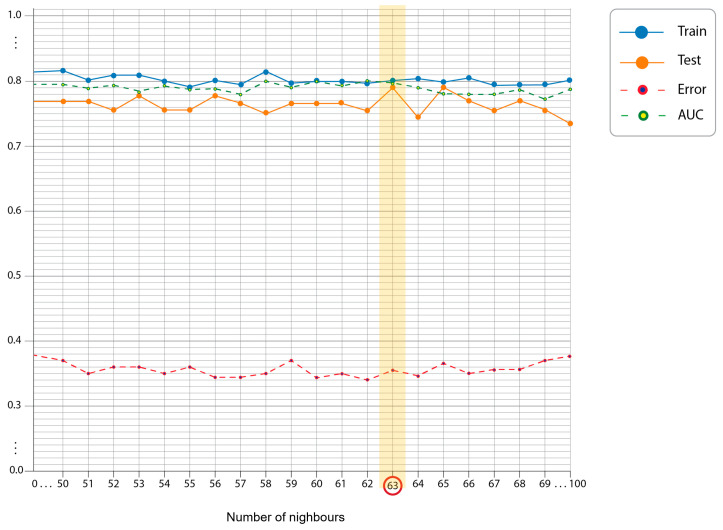
The optimization learning curves for the kNN model (distance measure Chebyshev). The optimal number of nearest neighbors in this case is 63, with a good fit represented by a small gap between the train and test learning curves, along with an acceptable error rate and its AUC value.

The optimization of hyperparameters for the ML models was independently performed for each model configuration using 10-fold cross-validation, with a focus on minimizing prediction error and maximizing AUC, while also taking into consideration the level of overfitting (Figures S1 and S2). An example of the hyperparameter selection process illustration is given in Figure 3. 

**Figure 4:**
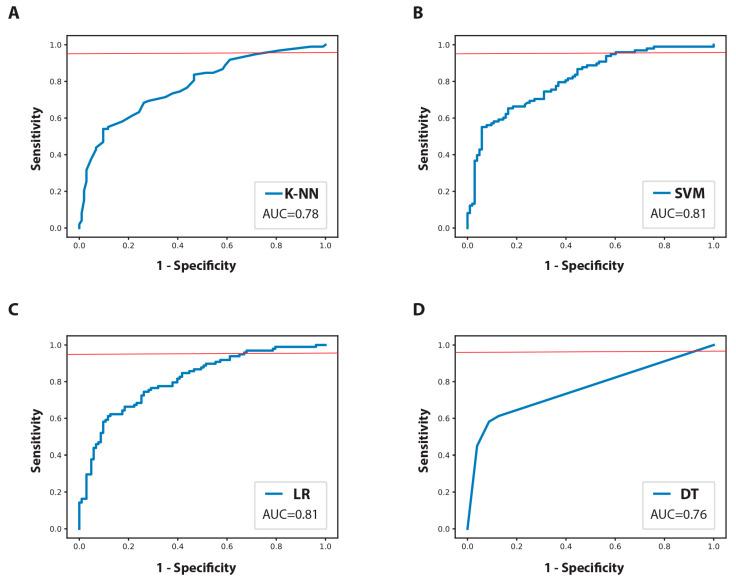
Receiver operating characteristic (ROC) curves for the training dataset. (**A**) k-Nearest Neighbors (KNN); (**B**) support vector machine (SVM); (**C**) logistic regression (LR); (**D**) decision tree (DT). The red line marks a 95% sensitivity cutoff. Abbreviation: AUC—area under receiving operating characteristics curve.

Thereafter, the ML models were evaluated for the various quality metrics. First, we compared the selected ML models based on their AUC values (Figure 4). The optimal probability output cutoff for each model was determined to achieve a minimum target sensitivity of 95% (Figure S3). The decision to prioritize high sensitivity was made in order to minimize the risk of failing to identify patients who might be at a higher risk of LNM and who later on can present in the form of recurrence if not provided with adequate treatment. 

The distribution of other performance metrics for the ML models in the training phase is given in Table 3. During the training phase, the kNN and SVM showed the best accuracy and F1 and F2 scores while the SVM and LR showed the best AUC. Among the evaluated models in this study, the decision tree model had the lowest values for every metric assessed, indicating the worst performance. To address concerns about the generalizability of the training models, we also analyzed overfitting learning curves (Supplementary Figures S1 and S2). Compared to kNN, our analysis revealed that the SVM and LR models exhibited a higher degree of overfitting. A model with high AUC but high overfitting can potentially overemphasize the noise present in the training data rather than capturing the actual underlying patterns. As a result, the model may perform suboptimally on new data.

The final ML classifier was obtained by selecting the one yielding the highest specificity and the lowest degree of overfitting while maintaining a sensitivity of 95%. Among the models evaluated, the kNN classifier was found to meet these criteria and was deemed the best-performing model. 

**Figure 5:**
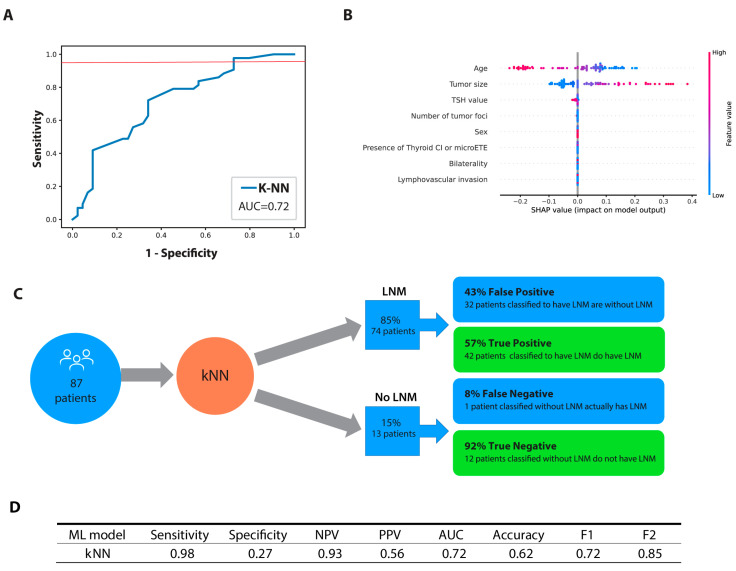
Final classifier performance. (**A**) Receiver operating characteristic (ROC) curve for the test dataset; (**B**) Beeswarm plot of SHAP weights of the model features for each patient; (**C**) Flowchart and confusion matrix showing performance of the final k-Nearest Neighbor (k-NN) model on the test data set; (**D**) The overall performance metrics of the final k-Nearest Neighbor model in the test phase. Abbreviations: TSH—thyroid stimulating hormone; CI—capsular invasion; ETE—microscopic extrathyroidal extension; NPV—negative predictive value; PPV—predictive positive value; AUC—area under receiving operating characteristics curve; F1 score; F2 score.

Subsequent testing on the kNN classifier on a test group of 87 patients revealed an AUC of 0.72, a sensitivity of 0.98, and a specificity of 0.27. While the model achieved an F1 score of 0.71 based on the harmonic mean of positive predictive value and sensitivity, our study places a greater emphasis on sensitivity. In this regard, the F2 score, which gives more weight to sensitivity, is more important in our study. The model achieved an F2 score of 0.85, indicating strong performance in terms of sensitivity. The kNN model predicted that 74 patients had lymph node metastasis, out of which 42 (57%) were accurately identified based on pathological confirmation. Among the 13 patients who were predicted by the model to have no lymph node metastasis, one patient (8%) would have been incorrectly classified as node-negative. The performance of the kNN model with the test cohort is summarized in Figure 5A,C,D.

To assess the variable importance, SHAP values were used and a beeswarm plot (Figure 5B) was generated to display the SHAP values for each feature across all patients. The analysis revealed that age and tumor size were the most important features in predicting LNM, with younger age and larger tumor size being positively associated with LNM. 

### 3.4. Web-Based Calculator

A decision-support web application based on a sensitivity-optimized kNN-machine learning model was created to predict the potential of cervical LNM by inputting patients’ demographic and histopathological characteristics. The calculator can be accessed using the following link (http://109.92.182.91:8089 (accessed on 26 April 2023)).

## 4. Discussion

In this study, we evaluated and compared four ML classifiers to predict central and lateral LNM in clinically node-negative T1 and T2 PTC patients by incorporating their clinical and histopathological characteristics. Our findings indicated that the kNN classifier had the highest sensitivity and therefore showed the potential to be used clinically to enable the identification of patients at a higher risk of LNM. Moreover, we developed an accessible web-based calculator to facilitate the practical implementation of the kNN classifier by inputting relevant clinical and histopathological characteristics of PTC patients, providing a useful tool for predicting LNM risk.

There is currently no universally accepted surgical approach to treat clinically node-negative PTC patients. The 2015 ATA guidelines stated that thyroidectomy without prophylactic LND is sufficient for small, cN0 PTC cases [9]. Nevertheless, the likelihood of nodal metastasis among this group is relatively high, with central LNM occurring in 16–53% of cases while 18.6–39.5% of PTC patients may present with occult lateral LNM [4,24]. Our data are in line with these findings, with 49% of patients having metastases in their lymph nodes, of whom 41.4% showed evidence of central lymph node involvement and 25% presented a lateral LNM. Such results imply a failure of preoperative diagnostics to detect patients with LNM which may later present as a persistent or recurrent disease requiring reoperation. Therefore, a more sensitive diagnostic method based on real clinical data is necessary to assist physicians to perform a more patient-centered postsurgical follow-up program. Improved LNM prediction can enable appropriate postsurgical treatment for patients in higher risk groups while also avoiding or minimizing unnecessary treatment for those at lower risk.

Our study considered eight clinical and histopathological characteristics as possible indicators for cervical LNM. The pretreatment variables assessed included age, sex, and TSH level. From our study’s results, there was a significant tendency for LNM to appear in younger patients. This is consistent with findings from other studies that being <45 years old is associated with a higher risk of LNM [25,26,27]. The SHAP values obtained from the KNN model further support this result when age was ranked first in terms of feature importance. The univariate analysis found no significant differences in the TSH values and sex between LNM and non-LNM patients. However, the SHAP values point to TSH being a potential predictor of LNM. The unexpected finding of lower TSH values associated with higher incidences of LNM might be due to confounding factors related to the unequal distribution of TSH values between the training and test groups. This would suggest that the impact of TSH levels is still not fully understood and further research is needed.

Our research also examined the impact of intraoperative and post-treatment characteristics on the development of LNM through analysis of tumor size, multifocality (number of tumor foci), bilaterality, micro ETE, thyroid CI, and LVI. Tumor size is considered to be an important factor for LNM [6,27,28]. We showed that the cervical LNM was found to relate positively to the primary tumor size (i.e., as the tumor increased in size, incidences of cervical lymph node metastasis also increased). Our PTC study confirms multifocality and bilaterality, previously reported as risk factors for lymph node metastasis, are indeed associated with the higher incidence of LNM [6,27,29]. We further extended our research to explore the association between the number of tumor foci and the incidence of LNM. We found that an increase in the number of tumor foci had a direct correlation to the likelihood of increase in LNM which is consistent with the results of other studies [30,31]. Our results found that microscopic ETE and thyroid CI may significantly associate with LN metastases, consistent with previous research [25,32]. This may be explained by the presence of rich lymphatic tissue around the thyroid which allows the direct transfer of the tumor cells into the lymph nodes [26]. Similar to other studies, our findings demonstrated that the presence of LVI in PTC patients is a significant predictor of LNM, as tumor cells may disseminate through lymphovascular spaces resulting in metastases [25,33]. While the univariate analyses suggested several variables to be significant LNM predictors, the SHAP analysis revealed only tumor size as the important one. This may be explained by the significance of the other variables’ effects on LNM being overshadowed by a tumor size’s strong effect. Therefore, to evaluate the significance of multiple and diverse variables as predictors of LNM, it is important to consider both univariate and SHAP analysis.

Preoperative evaluation and staging are most commonly based on US examination. According to meta-analyses conducted in 2019 and 2022, US has a sensitivity ranging from 28 to 33% for detecting central LNM, while being more sensitive in detecting lateral LNM (70 to 73%) [24,34]. If prophylactic LND is not performed, occult LNMs often remain undetected, leading to the classification of patients in the low or intermediate ATA risk group, for which additional RAI therapy is generally not required. Nevertheless, improved LNM prediction in PTC patients could lead to more personalized patient management, potentially replacing the current universal follow-up plans with more tailored surveillance. For instance, in the last years, additional functional imaging with a postoperative, pre-ablation diagnostic radioiodine whole body scan (DxWBS) was recommended, allowing for patients to be staged more precisely [35,36]. DxWBS with single-photon emission computed tomography with a computed tomography (SPECT/CT) scan might be able to provide detection for LNM of normal size which could not be adequately accessed on a preoperative neck ultrasound [36]. As Figure 5C illustrates, out of the 87 patients, 71 of them (85%) would have been predicted to have LNM and among those predicted cases, more than half of them, 42 patients (57%), were confirmed to have pathologic LNM that had been missed by the preoperative US examination. If we were to perform DxWBS with SPECT/CT on patients in whom the kNN model predicted LNM, it might modify their management, potentially resulting in additional surgery, a recommendation for RAI treatment, or changes to the prescribed RAI dosage [36]. Moreover, due to its high sensitivity, the kNN model prediction could be utilized as a complementary method to postoperative ultrasound, which has notably higher specificity, enabling a more individualized follow-up in medical centers where DxWBS with SPECT/CT is unavailable. On the other hand, among the 13 patients who were pathologically confirmed to be node-negative, the model incorrectly classified 1 patient. This means that for more than 90% of patients in whom the model predicted no LNM, close follow-up with DxWBS is not necessary and regular check-ups might be sufficient for such patients. Nevertheless, as with all predictive models, the price of increased sensitivity is a decrease in specificity. Given the above, 32 patients (42%) would be recommended for DxWBS with SPECT/CT without any benefit for patients. However, given the advantages of DxWBS with SPECT/CT in patients who have an occult LNM, a minor exposure to radiation can be deemed insignificant.

To date, few ML models have been constructed to predict LNM in PTC patients. Their results indicated that ML models have the potential to predict which patients may be at a higher risk of LNM with some of the studies demonstrating the ability of the ML classifiers to outperform the US in terms of predictive accuracy [18,37,38,39]. Although showing positive results, these studies included patients already suspected of LNM detected through preoperative assessment, while our study included only clinically node-negative PTC patients. Comparable to our research, a study from Feng et al. demonstrated that ML models are applicable to aid personalized predictions of central LNM in cN0 PTC patients. The performance of ML classifiers was assessed through AUC and ranged from 0.69 to 0.86 [28]. Nevertheless, their study encompassed a patient population with tumors exceeding 40 mm in size who were already known to be at a higher risk for developing LNM and for which prophylactic LND was already suggested by the guidelines. Unlike their study, our investigation focused exclusively on a cohort of clinically node-negative T1 and T2 PTC patients, similar to the study conducted by Zhu et al. [6]. Whereas their model only predicted central LNM, we investigated the accuracy of the ML model to predict central and lateral LNM in the same subgroup of patients. Regarding the performance metrics evaluated, the additional benefit of our study was that we incorporated metrics such as F1 and F2 scores. The aim of our study was to reduce the number of FNs in order to avoid missing patients who had LNM and who may potentially experience recurrence. Since accuracy does not provide information on whether there are more FNs or FPs, relying solely on this metric may not be useful for adequate diagnosis. Therefore, when interpreting results, it is necessary to also incorporate the F scores that more comprehensively summarize the confusion matrix [40].

Our work has several limitations. The retrospective nature of data collection might have resulted in selection bias and precluded the assessment of certain relevant risk factors for the development of LNM. Moreover, the identification of lateral LNM was established by sentinel lymph node analysis, which is limited by a relatively high false-negative ratio. This can result in patients with metastases being falsely classified as node-negative. Furthermore, in our study, the presence/absence of LNM was defined as the output variable without distinguishing between central and lateral lymph nodes. Therefore, a more detailed analysis of the factors that influence the appearance of one of these two types of LNM is needed to enable more precise stratification. Finally, by including only patients treated within our institution, the general applicability of the ML model may be limited.

### Future Perspectives and Outlook

In order to improve the prediction of ML models, integration of additional parameters, such as preoperative CT and US image features, will have significant role in the future. Incorporating image data can provide more detailed information and improve the sensitivity and specificity of the prediction models, enabling more accurate estimation of cervical LNM. Furthermore, the incorporation of deep learning models should be explored, utilizing their ability to learn directly from raw data, thereby potentially discovering complex patterns and features leading to improved prediction performance. To improve the generalizability and clinical applicability of the ML models, multicentric, external validation studies utilizing larger prospective patient cohorts should be applied. These efforts will strengthen the reliability and practical implementation of the ML models in real-world clinical settings.

## 5. Conclusions

The ML classifiers demonstrate potential for application in clinical practice to predict LNM and guide patient-oriented follow-up. Earlier detection of LNM could be crucial for appropriate risk stratification and timely interventions such as radioactive iodine treatment. In cases where standard diagnostic modalities yield negative results but the classifier indicates a higher likelihood of LNM, functional imaging could be introduced and followed by active surveillance or further treatment when necessary. Nonetheless, further clarification and optimization, including additional imaging parameters and high-quality data, are essential to enhance their performance and enable their full integration into clinical decision-making.

## Figures and Tables

**Table 2:** Comparison of patient characteristics by lymph node metastasis status.

Charasteristics	LNM−	LNM+	*p* Value
	(*n* = 147)	(*n* = 141)	
Age	50.7 ± 12.9	43.2 ± 13.1	<0.001
Sex			
Male	35 (23.8)	37 (26.2)	0.634
Female	112 (76.2)	104 (73.8)	
TSH value (µIU/mL)	1.67 (0.01–11.9)	1.80 (0.01–10.3)	0.255
Tumor size (in mm)	7 (1–40)	14 (1–40)	<0.001
Tumor size (categories, mm)			
≤5	46 (31.3)	11 (7.8)	
6–10	62 (42.2)	37 (26.2)	<0.001
11–20	30 (20.4)	56 (39.7)	
21–40	9 (6.1)	37 (26.2)	
Multifocality			
No	103 (70.1)	81 (57.4)	0.026
Yes	44 (29.9)	60 (42.6)	
Number of tumor foci			
1	103 (70.1)	81 (57.4)	
2	34 (23.1)	82 (19.9)	0.004
≥3	10 (6.8)	32 (22.7)	
Bilateral			
No	117 (79.6)	95 (67.94)	0.019
Yes	30 (20.4)	46 (32.6)	
Presence of Thyroid CI or microETE			
No thyroid CI or micro ETE	107 (72.8)	74 (52.5)	
Thyroid CI	24 (16.3)	34 (24.1)	<0.001
MicroETE	16 (10.9)	33 (23.4)	
Lymphovascular invasion			
No	144 (98.0)	129 (91.5)	0.014
Yes	3 (2.0)	12 (8.5)	

Values are presented as median (minimum–maximum), mean (standard deviation), or number (%) of patients. Abbreviations: −—negative; +—positive; LNM—lymph node metastasis; TSH—thyroid stimulating hormone; micro ETE—microscopic extrathyroidal extension.

**Table 3:** The overall performance metrics of the classifiers in the training phase.

ML Model	Sensitivity	Specificity	NPV	PPV	AUC	Accuracy	F1	F2
KNN	0.95	0.28	0.85	0.56	0.78	0.61	0.70	0.83
SVM	0.98	0.27	0.93	0.56	0.81	0.62	0.71	0.85
LR	0.98	0.21	0.91	0.54	0.81	0.59	0.69	0.84
DT	0.95	0.09	0.67	0.50	0.76	0.51	0.66	0.81

Abbreviations: kNN—k-Nearest Neighbors; SVM—support vector machine; LR—logistic regression; DT—decision tree; NPV—negative predictive value; PPV—predictive positive value; AUC—area under receiving operating characteristics curve; F1 score; F2 score.

## Data Availability

The datasets used and analyzed during the current study, as well as the Python script, are available from the corresponding author upon reasonable request.

## Supplementary Materials

The following supporting information can be downloaded at: https://zenodo.org/record/7864130#.ZEgNA85BxPY (accessed on 26 April 2023), Figure S1: Performance comparison and fine tuning of the k-Nearest Neighbor (kNN) classifiers and their distance metrics on the training dataset; Figure S2: Performance comparison and hyperparameter tuning of the machine learning classifiers on the training dataset; Figure S3: Sensitivity and specificity plots for machine learning classifiers on the training dataset; Table S1: Comparison of patients’ demographic and clinicopathological characteristics between training and test sets.
